# Editorial: Prognostic value of inflammatory and thrombotic biomarkers in acute coronary syndrome

**DOI:** 10.3389/fcvm.2026.1814669

**Published:** 2026-04-07

**Authors:** Danilo Menichelli, Laura Gatto, Flavio Giuseppe Biccirè

**Affiliations:** 1 Department of General Surgery, Surgical Specialty and Anesthesiology, Sapienza University of Rome, Rome, Italy; 2 Cardiovascular Sciences Department, San Giovanni Addolorata Hospital, Rome, Italy; 3 Centro per la Lotta Contro L’Infarto—CLI Foundation, Rome, Italy

**Keywords:** ACS, acute coronary syndrome, biomarkers, inflammation, thrombotic

Ischemic heart disease remains the leading cause of death and disability-adjusted life-years (DALYs) worldwide, especially among patients over 50 years of age (1).

Despite substantial advances in pharmacological and interventional management, acute coronary syndrome (ACS) is still associated with significant morbidity and mortality, and in-hospital and post-discharge complications are frequent (2). The pathogenesis of ACS is complex and multifactorial, involving both inflammatory and non-inflammatory mechanisms that contribute to plaque destabilization, including plaque rupture or erosion (3). These processes ultimately lead to the formation of a fibrin-rich thrombus and acute coronary obstruction, resulting in myocardial ischemia and necrosis.

Therefore, identification and validation of prognostic biomarkers in the context of ACS represent a critical step toward improving risk stratification and enabling the early identification of patients at increased risk of adverse outcomes. Biomarkers reflecting inflammatory activation, metabolic stress, and thrombotic processes may provide valuable insights into disease severity and progression and may help guide therapeutic decisions and patient management.

For this reason, the *Frontiers in Cardiovascular Medicine* Research Topic entitled “*Prognostic Value of Inflammatory and Thrombotic Biomarkers in Acute Coronary Syndrom*e” aims to identify several inflammatory and thrombotic biomarkers that could serve as a diagnostic and prognostic tool for clinicians. The Research Topic consists of eight articles that collectively advance this field.

Among inflammatory biomarkers, Kou et al. investigated the role of C-reactive protein (CRP) and interleukin 38 (IL-38), one of the latest members identified in the IL-1 cytokine family, as predictors of major adverse cardiac events (MACEs). MACEs are a severe complication often occurring after a percutaneous coronary intervention (PCI): indeed, while inflammation, as indicated by high serum CRP levels, was strongly associated with a higher risk of MACEs in post-PCI patients, IL-38 levels were inversely related to MACEs, probably due to the ability to bind to the IL-36 receptor (IL-1R6), which exhibits anti-inflammatory properties.

Xiong et al. examined the acute predictive value of monocyte count, monocyte-to-white blood cell ratio, and monocyte-to-lymphocyte ratio for severe heart failure in 173 patients with acute myocardial infarction. The authors found that these biomarkers are significantly higher in patients with acute severe heart failure, representing potential biomarkers for this complication.

Yang et al. investigated the role of the Systemic Inflammatory Response Index (SIRI), defined as [(neutrophil count × monocyte count)/lymphocyte count], in predicting myocardial infarction in patients with unstable angina. Indeed, unstable angina can rapidly progress to myocardial infarction, and inflammation plays a key role in this process. For this reason, the authors investigated SIRI as a potential predictor of myocardial infarction in 129 patients with unstable angina. In this study, SIRI demonstrated excellent predictive performance for myocardial infarction, resulting in an independent predictor of myocardial infarction in this population.

Beyond inflammation, metabolic stress and glycemic abnormalities are additional determinants of adverse outcomes in ACS. Wang et al. evaluated the stress-hyperglycemia ratio (SHR) as a potential predictor of in-hospital cardiogenic shock in 1,776 patients with ST-elevation myocardial infarction (STEMI). The authors found that SHR is independently associated with in-hospital cardiogenic shock, and including this ratio in established risk scores significantly improves predictive accuracy.

Wang et al. performed a cross-sectional study of 264 patients with STEMI and 264 healthy controls, evaluating whether serum cyclophilin A (CyPA), a novel biomarker, could be helpful in prognosis improvement. The authors found that serum CyPA levels are significantly higher in patients with acute STEMI than in healthy controls and are independently associated with acute STEMI, suggesting a potential role for this biomarker in the identification of STEMI in the early stages.

Miéville et al. conducted a preclinical study on pigs to evaluate the role of the FnBPA5 peptide as a potential microthrombi biomarker in patients with Myocardial Infarction with Non-Obstructive Coronary Arteries (MINOCA), which accounts for up to 15% of acute myocardial infarctions. As shown by this study, FnBPA5 appears to enable the selective detection of microthrombi in coronary arteries by targeting untensed fibronectin fibers, a novel mechanical biomarker of microthrombi, making this biomarker a promising tool for advanced microthrombi detection.

Li et al. explored the correlation between the Gensini score, which quantifies coronary artery lesions, and fasting blood glucose (FBG) levels in 464 STEMI patients who underwent PCI. The U-shaped relationship found a significant, non-linear association between FBG levels and coronary artery lesion severity, regardless of diabetes status, suggesting a potential role for FBG monitoring in STEMI patients.

Finally, Pannunzio et al. summarized the two-way relationship between atrial fibrillation (AF) and coronary artery disease in a narrative review: indeed, these disorders often coexist and may represent a clinical feature that could predict the other condition. In particular, AF and acute myocardial infarction share several mechanisms of injury, such as increased oxidative stress, systemic inflammation, and increased platelet aggregation. Furthermore, the authors described the pathophysiological differences between early AF (occurring within the first 24 h after the index event and associated with atrial ischemia, oxidative stress and a better outcome) and late AF (occurring after 24 h from myocardial infarction and correlated with increased left atrial pressures, deterioration of hemodynamic status, and a worse outcome) suggesting that the timing of AF onset may itself represent a clinically relevant prognostic marker of a worse outcome in patients with acute myocardial infarction.

In conclusion, the studies included in this Research Topic emphasize the central role of inflammatory activation, metabolic stress, and thrombotic mechanisms in determining disease severity and clinical outcomes in ACS. Emerging biomarkers reflecting these biological pathways provide valuable prognostic information and may contribute to improved risk stratification and personalized management of patients with ACS.
